# SARS-CoV-2 and Pre-existing Vascular Diseases: Guilt by Association?

**DOI:** 10.1177/11795468211010705

**Published:** 2021-05-16

**Authors:** Grigorios Voulalas, Janice Tsui, Luciano Candilio, Daryll Baker

**Affiliations:** 1 Vascular Surgery Department, Royal Free London NHS Foundation Trust, London, UK; 2 Division of Surgery & Interventional Science, University College London, UK; 3 Cardiology Department, Royal Free London NHS Foundation Trust, London, UK

**Keywords:** Arterial thrombosis, venous thromboembolism, severe acute respiratory syndrome coronavirus-2, coronavirus disease-19

## Abstract

Severe Acute Respiratory Syndrome coronavirus-2 has rapidly spread and emerged as a pandemic. Although evidence on its pathophysiology is growing, there are still issues that should be taken into consideration, including its effects on pre-existing peripheral vascular disease. The aim of this review is to describe the thrombotic and endothelial dysfunctions caused by SARS-CoV-2, assess if cardiovascular comorbidities render an individual susceptible to the infection and determine the course of pre-existing vascular diseases in infected individuals. A search through MEDLINE, PubMed and EMBASE was conducted and more than 260 articles were identified and 97 of them were reviewed; the rest were excluded because they were not related to the aim of this study. Hypertension, cardiovascular disease, diabetes mellitus and cerebrovascular diseases comprised 24.30% ± 16.23%, 13.29% ± 12.88%, 14.82% ± 7.57% and 10.82% ± 11.64% of the cohorts reviewed, respectively. Arterial and venous thrombotic complications rocketed up to 31% in severely infected individuals in some studies. We suggest that hypertension, cardiovascular diseases, diabetes and cerebrovascular diseases may render an individual susceptible to severe COVID-19 infection. Pre-existing vascular diseases are expected to deteriorate with SARS-CoV-2 infection as a consequence of its increased thrombotic burden and the development of endothelial dysfunction. COVID-19 has emerged only a few months ago and it is premature to predict the long-term effects to the vascular system. Its disturbances of the coagulation mechanisms and effects on vascular endothelium will likely provoke a surge of vascular complications in the coming months.

## Introduction

Coronavirus disease 2019 (COVID-19) is caused by the severe acute respiratory syndrome coronavirus 2 (SARS-CoV-2) and has rapidly spread around the globe and emerged as a pandemic. Although evidence on its pathophysiology and the identification of the most vulnerable populations is growing, its effects on pre-existing peripheral arterial and venous disease is undetermined yet.

The National Centre for Immunization and Respiratory Diseases (NCIRD) has proposed the risk factors that render an individual susceptible to COVID-19 infection (https://www.cdc.gov/coronavirus/2019-ncov/need-extra-precautions/people-at-higher-risk.html) but how this virus is related to the deterioration of a pre-existing venous and arterial disease is still unknown. A recognized observation is that the virus shares common potential risk factors with cardiovascular diseases, and the severity of its course may be associated with thrombotic complications.

In the current review, we aim to assess if cardiovascular comorbidities and diabetes mellitus (DM) render an individual at risk for COVID-19 and mortality, refer to the thrombotic complications and endothelial dysfunctions caused by the virus, identify the course of pre-existing vascular diseases in COVID-19 patients and describe their prognosis.

## General Considerations, Mechanism of Action and Risk Factors

COVID-19 is caused by SARS-CoV-2: the virus is mainly transmitted by droplets generated in the airway of an infected individual and is ejected through coughing, sneezing and talking.^1^ The viral loaded droplets can enter the host through the epithelial cells of the upper respiratory tract and the conjunctiva. Another potential route of transmission is through the gastrointestinal tract^1^; the virus can survive on surfaces and its infective potential through this route should not be underestimated.^1,2^

The virus uses a glycoprotein on its surface (S1) which has high affinity to angiotensin-converting enzyme type 2 (ACE2) receptors in order to invade cells.^1,3^ These receptors are interspersed in different human tissues; therefore, the virus has the capacity to infect different cell types and induce different pathogenic pathways with variations in clinical picture.^1^ ACE2 is a membrane-bound monocarboxypeptidase expressed predominantly in heart, intestine, kidney and pulmonary alveolar type II cells.^3^ In addition, ACE2 receptor is expressed in vascular endothelial cells.^4^ ACE-2 dependent SARS-CoV-2 entry can be blocked by an inhibitor of cellular serine protease TMPRSS2, which is employed by the virus for S protein priming.^5^ Other proteins involved in S protein priming include the endosomal cysteine proteases cathepsin B and L (CatB/L).^5,6^ TMPRSS2 is a cell surface protein expressed by epithelial cells within the airway and alveolar spaces,^7^ which facilitates the fusion of SARS-CoV-2 and cellular membranes.^8^ Single cell RNA-sequence expression profiling studies have showed that it is mostly expressed in alveolar type II cells, nasal airway epithelial cells, digestive system, brain tissue, the heart and the kidneys.^9^ Furthermore, one more receptor that could be implicated in SARS-CoV-2 cellular invasion is transmembrane dipeptidylpeptidase 4 (DPP4), but crystallographic studies showed that the binding affinity was low for SARS-CoV-2.^10^ DPP4 is expressed in kidneys, intestine, liver, thymocytes, pancreas, lungs, in the cells of the haematopoietic lineage and in the capillary beds, mainly in the endothelium of the venules.^11^ COVID-19 pathogenicity depends on its ability to overcome the host’s immune system-both innate and adaptive.

According to NCIRD, individuals who are at higher risk for severe illness from COVID-19 are those aged > 65 years and older, who live in nursing homes or long-term care facilities, the immunocompromised, or individuals with concomitant diseases such as chronic lung disease, heart disease, type 1 or 2 DM, liver disease, chronic kidney disease on dialysis and those with severe obesity (Body Mass Index ⩾ 40 kg/m^2^) (https://www.cdc.gov/coronavirus/2019-ncov/need-extra-precautions/people-at-higher-risk.html).

Whilst this review focuses on cardiovascular and thromboembolic risk factors and complications of COVID-19, diabetes is key risk factor for cardiovacular diseases and impacts on their outcomes. Therefore its effect on COVID19 infection is also considered here.

## Are Patients with Pre-existing Cardiovascular Diseases and/or DM More Vulnerable to COVID-19 Infection or Severe/Mortal COVID-19 Infection?

Huang et al in a retrospective study reported that 20% of the infected patients had DM, 15% hypertension and 15% pre-existing cardiovascular diseases,^12^ while Chen et al reported 40% of concomitant cardiovascular and cerebrovascular diseases in their cohort.^13^ Similarly, Zhou et al reported that 30% of patients in their cohort had pre-existing hypertension, 19% DM and 8% coronary heart disease^4^ and Wang et al reported that from a total of 138 patients admitted with COVID-19, 31.2% had hypertension, 14.5% cardiovascular disease, 10.1% DM and 5.1% pre-existing cerebrovascular disease.^14^ Inciardi et al included 99 patients infected with COVID-19 and reported that concomitant DM, cardiovascular diseases, hypertension and cerebrovascular diseases were present in 31%, 52%, 64% and 30% of individuals, respectively.^15^ The comorbidities described are shown in Table 1.

**Table 1.** Demographic characteristics of patients infected with COVID-19 in different studies.

Study/number of patients included	Diabetes mellitus (%)	Cardiovascular diseases (%)	Hypertension (%)	Cerebrovascular diseases (%)
Huang et al^12^ (n = 41)	20	15	15	-
Zhou et al^4^ (n = 191)	19	8	30	-
Wang et al^14^ (n = 138)	10.1	14.5	31.2	5.1
Yang et al^16^ (n = 52)	17	10	-	13.5
Wu et al^17^ (n = 201)	10.9	4	19.4	-
Guan et al^18^ (n = 1099)	7.4	2.5	15	1.4
Kui et al^19^ (n = 137)	10.2	7.3	9.5	-
Liu et al^20^ (WHO data)	7.3	10.5	6.0	-
Inciardi et al^15^ (n = 99)	31	52	64	30
Yan et al^21^ (n = 193)	24.9	16.1	37.8	4.1
Yang et al^22^ (n = 1576)	9.7	8.4	21.1	-
Cao et al^23^ (n = 46,959)	10.3	11.2	18.3	-
Mean value ± SD	14.82 ± 7.57	13.29 ± 12.88	24.30 ± 16.23	10.82 ± 11.64

It is obvious that about 1/7 of patients with COVID-19 had either DM or pre-existing cardiovascular disease and 1/10 had cerebrovascular disease, whereas ¼ had hypertension. From the available data we cannot safely conclude that patients with any of the aforementioned risk factors are at increased risk for COVID-19 virus infection. Even in the case of hypertension, the global prevalence is 22% according to WHO official site, https://www.who.int/images/default-source/infographics/ncds/prevalence.jpg?sfvrsn=3472e76c_2, so a mean value of approximately of 24% does not necessarily indicate increased risk for COVID-19 infection.

Obesity has been described as a main risk factor for COVID-19 vulnerability and mortality, but none of the studies depicted in Table 1 included obesity in their cohorts. A French study reported that the risk for mechanical ventilation in patients with COVID-19 infection was 7-fold increased for those with Body Mass Index (BMI) >35 compared with those who had BMI < 25 kg/m^2^.^24^

Nevertheless, the prevalence of the aforementioned risk factors in severe/mortal cases of COVID-19 has been described. In a retrospective longitudinal, multi-centred study from a cohort of 7337 confirmed COVID-19 cases, Zhu et al reported 13% prevalence of DM; however, in-hospital mortality rate was significantly higher in patients with pre-existing DM related to the non-diabetic individuals (7.8% vs 2.7%, *P* < .0010), while hazard ratio in the diabetics versus the non-diabetics was 2.90 (95% CI: 2.21-3.81, *P* < .001).^25^

In a meta-analysis with 3027 confirmed cases of COVID-19, the proportion of DM and pre-existing cardiovascular disease was statistically significantly higher in the critical/fatal group than the in non-critical/non-fatal group (DM: odds ratio [OR] = 3.68, 95% CI [2.68, 5.03], *P* < .00001;cardiovascular disease: OR = 5.19, 95% CI [3.25, 8.29], *P* < .00001); similarly they reported a higher proportion of hypertension in critical/fatal group (OR = 2.72, 95% CI [1.60, 4.64], *P* = .0002). Therefore, mortality was shown to be increased among individuals with the aforementioned pre-existing diseases.^26^

Furthermore, in another study conducted by Shi et al which included 1561 confirmed COVID-19 patients, the prevalence of diabetes was 9.8%, while multivariable Cox regression analyses of 306 patients (153 with DM and 153 age and sex matched without DM) showed that hypertension (hazard ratio 2.50, 95% CI: 1.30-4.78) and cardiovascular disease (hazard ratio 2.24, 95% CI: 1.19-4.23) were independently associated with in-hospital mortality, while diabetes was not significantly associated with mortality (hazard ratio 1.58, 95% CI: 0.84-2.99).^27^

Similarly, in a systematic review and meta-analysis, cerebrovascular and cardiovascular diseases were associated with increased risk for poor outcome in patients infected with COVID-19.^28^ Also, in a meta-analysis by Aggarwal et al, a previous history of cardiovascular diseases was associated with an 11-fold increase in mortality in patients with COVID-19.^29^ Similarly, Ruan et al associated the increased mortality risk with cardiovascular diseases in COVID-19 patients.^30^ In addition, Yan et al concluded that patients with diabetes had higher mortality rates than patients without diabetes (81.3% vs 47.6%) and more diabetics were admitted to the Intensive Care Unit (ICU).^21^

Similarly, when Yang et al compared the comorbidities between severe and non-severe patients, they found the odd ratios for hypertension and cardiovascular disease were 2.36 (95% CI: 1.46-3.83) and 3.42 (95% CI: 1.88-6.22), respectively, suggesting that hypertension and pre-existing cardiovascular diseases could be risk factors for severe COVID-19.^22^ In a meta-analysis including 1527 COVID-19 patients, hypertension accounted for 28.8% of severe cases and 14.1% of non-severe cases (risk ratio 2.03 [95% CI: 1.54-2.68]), cardiovascular diseases for 16.7% of severe cases, and 6.2% of non-severe cases (risk ratio 3.30 [95% CI: 2.03-5.36]) and DM accounted for 11.7% of severe cases, and 4% of non-severe cases (risk ratio 2.21 [95% CI: 0.88-5.57]).^31^ Therefore, patients with hypertension, cardiac and cerebrovascular diseases or DM are more likely to develop severe COVID-19.^31^ This finding was also confirmed by Cheng et al.^32^

While hypertension appears to be associated with more severe COVID-19, there is no strong evidence to indicate increased susceptibility of patients with hypertension to COVID-19.^33^ Therefore, we can conclude that DM, cardiovascular diseases, hypertension and cerebrovascular diseases do not necessarily render the individuals vulnerable to COVID-19 infection, but if these individuals are infected by COVID-19 virus then they have worse clinical outcomes. A summary of the above studies is shown in Table 2.

**Table 2.** Odds (OR) or hazard ratios (HR) for COVID-19 severity with respect to pre-existing diabetes, cardiovascular and cerebrovascular diseases, and hypertension.

Study/number of patients included	Diabetes	Cardiovascular diseases	Hypertension	Cerebrovascular diseases
Zhu et al^25^ (n = 7337)	2.90	-	-	-
Zheng et al^26^ (n = 3027)	3.68	5.19	2.72	-
Shi et al^27^ (n = 1561)	1.58	2.24	2.50	-
Yang et al^22^ (n = 1576)	-	3.42	2.36	-
Pranata et al^28^ (n = 4448)	-	2.23	-	2.38
Li et al^31^ (n = 1527)	2.21	3.30	2.03	-

Several mechanisms have been proposed to contribute to the worse prognosis in these individuals, with dysregulated immune responses being an important one. For example, in patients with obesity, changes in the innate and adaptive immune responses have been reported which affect the course of viral infections and determine their clinical outcome. Adipose tissue releases inflammatory cytokines, adipokines and angiogenetic molecules, while obese individuals present with leptin resistance.^34^ Zhang et al suggested that leptin resistance could worsen the outcome of patients during the H1N1 influenza A pandemic.^35^ Individuals with DM have abnormal host responses, including disorders of humoral immunity and defects in neutrophil function and T cell responses and impaired B cell functions.^36^ In hypertension, there have been observed altered profiles of pro- and anti-inflammatory cytokines and cytokine production capacity, while interleukin-6 (IL-6) increases blood pressure.^37^ Inflammation is an important driver in cardiac pathological responses to stress as well and individuals with cardiovascular diseases present with lymphocytopenia; lymphocytopenia triggered by SARS-CoV-2 infection can add to the cardiovascular burden in COVID-19 patients.^38^

Furthermore, differences in immunity response to SARS-CoV-2 infection between males and females have been described. Males have been shown to express more ACE2 receptors and this can play a role in the severity of the disease observed.^39^ Differences in male and female immunological responses as well as the role of oestrogen and testosterone in priming the ACE2 receptor sensitivity could explain the higher COVID-19 severity and mortality rate observed in males.^39^ Male sex was associa-ted with a more severe COVID-19 infection,^40^ and higher mortality.^41–44^ However, there are not sufficient data to correlate the severity of infection and mortality rate to the incidence of cardiovascular comorbidities in males. Meng et al conducted a retrospective analysis of 168 severe patients in Wuhan, China and concluded that although age and comorbidities were both important factors that influenced the progression of COVID-19, the impact showed pronounced sex-specific differences; male had a higher trend towards a higher risk of mortality and a lower hospital discharge rate (male vs female: cardiovascular disease 7% vs 3.7%; DM 12.8% vs 11.0%; hypertension 23.3% vs 25.6%).^45^

The risk factors of COVID-19 identified are similar to those seen in seasonal and pandemic influenza. Specifically, the history of prior cardiovascular diseases, DM, age and chronic lung diseases have been reported as risk factors for increased hospital admissions, intensive care admissions and death in seasonal and pandemic influenza, as analysed by Mertz et al in a systematic review.^46^ Specifically, increased age was associated with higher risk of admissions and death (odds ratio 2.95 [95% CI: 1.53-5.70]), DM was associated only with higher risk of hospital admission in seasonal influenza but with higher risk of death in pandemic influenza, and cardiovascular comorbidities were associated with increased risk of hospital admission and death both in seasonal and pandemic influenza (odds ratio 2.92 [95% CI: 1.76-4.86]).^46^ Therefore, influenza and COVID-19 share common risk factors for worse outcomes, and this fact should be taken into consideration when designing future vaccination and therapeutic measures.

## Are Patients with Pre-existing Cardiovascular Diseases and/or Diabetes More Vulnerable to COVID-19 Virus Related Cardiovascular Complications?

Complications caused by COVID-19 include myocardial injury (7%-17%)^4,12,14^; diagnosed if serum levels of cardiac troponin I were above the 99th percentile upper reference limit, or new abnormalities were shown in electrocardiography and echocardiography,^12^ myocarditis, heart failure, myocardial infarction, arrhythmias (16.7%),^14^ deep venous thrombosis (DVT)/pulmonary embolism (PE) (15%-23%).^15^ SARS-CoV-2 can cause myocardial injury by direct or indirect action.^33^ The direct injury is the result of the binding of the virus to the ACE2 receptors on cardiomyocytes, while the indirect action involves the cytokine storm that is elicited by the immune response to the infection or the oxygen supply imbalance induced by the acute respiratory distress syndrome (ARDS).^26^ Oudit et al reported that SARS-CoV viral RNA was detected in autopsied human heart samples suggesting direct myocardial invasion via ACE2 receptors^47^; the same mechanism has been suggested in SARS-CoV-2 infection.^33^ The detrimental effect of ACE2 downregulation would impede the cardioprotective effects of Ang 1 to 7, which would lead to increased tumour necrosis factor-α (TNF-α) production and subsequently to a severe inflammatory response.^47^ Guo et al found that heart failure samples displayed a substantial proportion of ACE2 positive cells in cardiomyocytes and high expression patterns of ACE2 and they suggested that SARS-CoV-2 may attack the heart through bloodstream.^48^ Another proposed mechanism is the rupture of the atheromatous plaques in coronary arteries by the circulating cytokines and the hypercoagulability provoked by the virus.^49,50^

There are currently not sufficient data to support the hypothesis that patients with pre-existing cardiovascular diseases, hypertension and diabetes are prone to increased risk of cardiovascular complications when infected with COVID-19 virus. Associations between cardiac troponin elevation and pre-existing cardiovascular diseases have not yet been performed in order to detect any evidence of causality.^33^ Inciardi et al reported that 17% of patients with pre-existing cardiac disease, such as heart failure, coronary artery disease and atrial fibrillation, developed venous thromboembolism (VTE), while 6% of them developed arterial thromboembolism: the overall mortality rate was 36% in patients with pre-existing cardiac disease compared with those with no cardiac disease (36% vs 15%, *P* .019) 36% vs 15%; P .019; relative risk 2.35; 95% CI: 1.08-5.09.^15^

Regarding the thromboembolic manifestations of COVID-19 virus, Klok et al reported that age and coagulopathy, defined as a spontaneous prolongation of the prothrombin time >3 seconds or activated partial thromboplastin time >5 seconds, were independent predictors of thrombotic complications.^51^ Furthermore, Lodigiani et al reported that the thromboembolic events occurred in the 7.7% of the hospitalized patients on wards and in the 16.7% of hospitalized patients on ICU, with a total cumulative rate of 21%.^52^ In addition, the prevalence of ischaemic stroke as a complication of COVID-19 virus was 2.5% in their cohort.^52^ However, neither Klok et al nor Lodigiani et al reported pre-existing cardiovascular diseases, diabetes or hypertension as predisposing factors for COVID-19 complications.^51,52^

For patients with underlying heart disease, SARS-CoV-2 infection might act as a precipitating factor to worsen the already existing disease and lead to death.^53^

## COVID-19 and Peripheral Venous and Arterial Complications

It has been described that COVID-19 triggers a coagulation response, leading to DVT and PE as well as thrombosis of microcirculation and disseminated intravascular coagulation (DIC). Ranucci et al reported a pro-coagulant profile in their cohort with increased IL-6, which induces tissue factor expression, high fibrinogen and d-dimer levels and prolongation of activated partial thromboplastin time (aPTT).^54^ D-dimer levels have been identified as markers of severity of COVID-19. Thromboembolic events occurred at a rate of 7.7% (95% CI 5.4-11%) and ischaemic stroke at 2.5% according to Lodigiani et al.^52^ Also, Inciardi et al reported rates of venous and arterial thromboembolism of 12% and 3%, respectively,^15^ while Griffin et al presented 3 cases of arterial thromboses in patients with COVID-19.^55^

The mechanisms by which COVID-19 virus induces thromboembolic events is still unknown. Severity of lung infection in combination with immobility, ageing, obesity, high fever and the systematic inflammatory response could potentially render the individuals susceptible to venous thromboembolic events. Features of DIC and PE are highly prevalent in COVID-19 patients.^33^ Local vascular microthrombosis in the pulmonary vascular tree can be the cause of in situ pulmonary artery thromboses, which further aggravates the respiratory function and increases mortality rates.

Fogarty et al found that severe COVID-19 infection was associated with significant coagulopathy that correlated with disease severity,^56^ while Klok et al suggested that the rate of thrombotic complications may be as high as 31% in the critically ill patients referring to both venous and arterial thromboses.^51^ Similarly, Middeldorp et al showed cumulative incidences of venous thromboembolism at 7, 14 and 21 days of 16%, 33% and 42%, respectively in their cohort including patients hospitalized on the wards and ICU.^57^ There were also referrals of acute aorto-iliac and mesenteric artery thrombosis in COVID-19 infected patients.^58^ Systemic inflammation, abnormal coagulation status, multiorgan dysfunction and severity of disease are contributing factors to the increased risk of venous thromboembolism.^59^ Additionally, Melissano et al reported 23% prevalence of DVT in severely ill COVID-19 patients, and stressed the increase of peripheral ischaemia prevalence in their centre.^60^

In addition, more evidence is rising in the literature regarding the arterial complications of COVID-19 virus in severely ill patients. There is increased anecdotal evidence of increased coronary thrombus burden in COVID-19 patients and, similarly, case reports of acute aortic and/or limb arterial thromboses have been reported with variable outcomes,^61–63^ further supporting the increased thrombotic risk of SARS-CoV-2 infection. He et al reported that 30% of the patients in their cohort had confirmed PE despite prophylactic anticoagulation therapy.^64^ A different pathologic finding was reported by Behzad et al; they described pulmonary arteries’ or subsegmental arteries’ enlargement and luminal dilatation in chest computed tomographic scans (CT) in COVID-19 patients, further enlightening the effect of the virus on vascular physiology.^65^ Their findings require further investigation in order to map the future impact of the virus to the development of arterial dilatation and possibly aneurysm formation.

The effect of COVID-19 infection on the cerebrovascular circulation should be taken into consideration. Sheraton et al reported both ischaemic and haemorrhagic strokes were described, with the ischaemic strokes being the commonest.^66^ Data showed that patients with severe systemic presentation and cardiovascular risk factors were more likely to have acute cerebrovascular diseases, while large vessel disease was the most common mechanism of the aetiology of strokes.^67^ In another study cerebrovascular disease was found to be associated with 2.5-fold increased odds of severe disease in patients with COVID-19.^68^ Oxley et al reported 5 cases of large vessel ischaemic stroke in patients younger than 50 years old who presented in New York Health Care systems, and suggested that the association between stroke and COVID-19 virus infections should be investigated.^69^ Two more cases of acute ischaemic stroke in COVID-19 patients following carotid artery thrombosis have been described.^70,71^ In a retrospective case series study, Mao et al reported that the prevalence of acute cerebrovascular disease in severe COVID-19 cases was 5.7%.^72^

Mechanisms of arterial thromboses such as direct viral endothelial target, hypercoagulability, endothelial dysfunction and uncontrolled inflammatory cascade have been proposed.^50^ High plasma levels of IL-2, IL-7, granulocyte-colony stimulating factor (G-CSF), interferon gamma induced protein 10 (IP10), monocyte chemoattractant protein 1 (MCP1), macrophage inflammatory protein 1A (MIP1A) and TNF-α have been observed in COVID-19 infected individuals.^73^ The experience with SARS-CoV has shown that it induces endoplasmic reticulum stress, lysosomal damage and cell death, while it activates inflammasome NLRP3.^1^ Endothelial dysfunction and high levels of von Willebrand factor (vWF), found in the plasma of COVID-19 individuals, play an essential role in the hypercoagulable state.^1,74^ Maier et al reported that all patients with severe COVID-19 (n = 15) had their plasma viscosity exceeding 95% of the normal as measured by capillary viscometry, adding one more piece of evidence of the hypercoagulable burden of the virus.^75^

Also, there have been reports of transient anticardiolipin IgA, anti-B2-glycoprotein IgA and IgG antibodies presence in COVID-19 patients provoking complex coagulopathy.^1,76^ Violi et alproposed the role of the enzyme Nox2, which is important in the generation of reactive oxygen species (ROS) and it is overactivated in patients with pneumonia.^77^ They suggested that Nox2 is implicated in artery dilatation and platelet activation by inactivation of nitric oxide (NO) or by enhancing eicosanoid production.^77^ SARS-CoV-2 infection is associated with oxidative stress, the proinflammatory state, cytokine production and cell death.^78^ Lower or impaired NO metabolism is associated with the pathological severity of COVID-19.^79^ NO synthesis requires oxygen and is thereby inhibited in ARDS due to hypoxia.^79^ In addition, one transcription factor that is involved in the homeostasis of ROS generation is Nuclear Factor Erythroid-derived 2-Related Factor 2 (Nrf2), whose activation has been reported to downregulate ACE2 expression and its deficiency to up-regulate it, while it protected cells from oxidative stress and inflammation.^80^

In a multi-centre prospective cohort study, Helms et al reported that vWF and vWF antigen were considerably increased in severe COVID-19 cases, as well as factor VIII (FVIII) and 87.7% of the patients had positive lupus circulating anti-coagulant during their hospitalization on ICU.^81^ In addition, severe COVID-19 cases were more often complicated with thromboses and PE.^81^ Furthermore, the hypothesis that the endothelium is a target of COVID-19 virus is gaining more support.^82^ ACE2 receptors are present in the endothelium; binding of the virus to its receptors might promote acute inflammation and hypercoagulation, mechanism which can explain the thrombotic state observed in COVID-19 patients.^82^ SARS-CoV-2 can infect endothelial cells triggering an immune response and ensuing activation of inflammation resulting in dysregulation of the endothelium, leukocyte activation, complement deposition and platelet consumption.^83^ In addition, angiotensin II levels in the plasma of COVID-19 patients were markedly elevated^82^; angiotensin II is known to increase microvascular permeability, induce transcription of tissue factor and activate platelets.^74^ Additionally, it can also trigger the release of several compounds of the complement system from endothelial cells establishing a pro-thrombotic state in COVID-19 positive patients.^74^ C5a and C5b-9 complement molecules were the key mediators of COVID-19 associated endothelial dysfunction as proposed by Noris et al.^84^ Also, localized macrophages can release plasminogen activators, whose production is affected by the activation of angiotensin II as well.^20^ Post-mortem studies have described the presence of viral inclusion structures in endothelial cells, presence of endothelitis and diffuse endothelial inflammation.^85^

**Figure 1.**
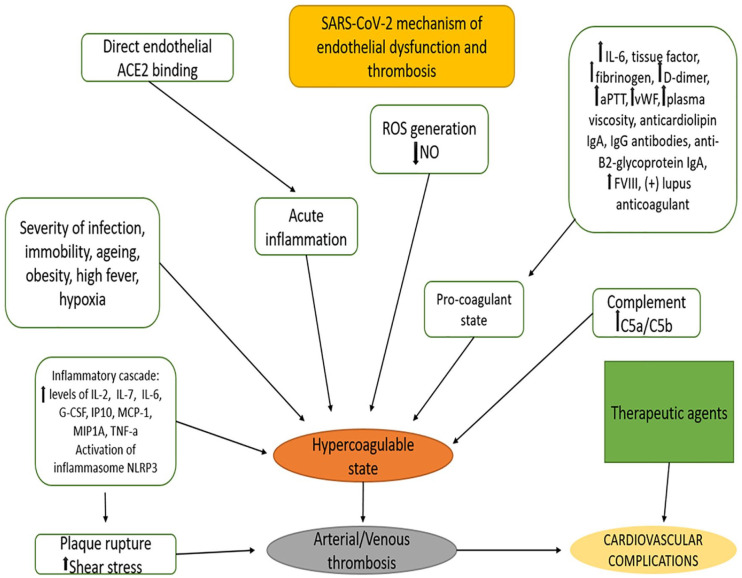
SARS-CoV-2 mechanisms of endothelial dysfunction and thrombosis.

Moreover, hypercoagulability is aggravated by hypoxia, which augments thrombosis by both increasing blood viscosity and by the activation of specific signalling pathways,^74^ such as Hypoxia-Inducible Factor (HIF) pathway.^86,87^ Another hypothesis that gains acceptance is the risk of atherosclerotic plaque rupture due to the systemic inflammation and the increased shear stress, and it has been described in the cases of myocardial infarction complications in COVID-19 patients. Many studies support this notion, which better explained the incidence of acute heart disease in infected individuals.^33,49,50,88,89^ The aforementioned mechanisms of endothelial dysfunction and thrombotic diathesis in COVID-19 are depicted in Figure 1.

## What Happens with COVID-19 Virus Infection and Pre-existing Peripheral Arterial and Venous Disease?

Although the risk factors for COVID-19 have been precisely described in studies, none refers to venous or arterial diseases in the periphery as risk factors and furthermore, none of the studies implicate pre-existing peripheral vascular disease in the severity of the infection or outcome, apart from cerebrovascular disease that was described both as a risk factor and its impact on outcome.^14–16,18,21,28,68^

Atherosclerosis and COVID-19 virus share common risk factors; age, diabetes, smoking, obesity and hypertension. Therefore, we can only assume that COVID-19 virus can deteriorate a pre-existing peripheral artery disease and limb ischaemia via either directly targeting the endothelium, as described before, or by introducing a hypercoagulable state. Stenotic arteries and diminished peripheral flow can be aggravated by platelet aggregates, increased blood viscosity and expression of coagulation factors such as vWF, fibrinogen and d-dimers.

Another possible mechanism of peripheral artery disease deterioration is the potential of atherosclerotic plaque rupture due to the cytokine storm that is elicited by the infection.^33^ Ruptured plaque can attract circulating platelets and lead to thrombus formation.^90^ In addition, several cytokines have been implicated in atherosclerosis development, while the activation of inflammasome NLRP3 is of paramount importance as well.^91^ COVID-19 virus may act through the same inflammasome,^1^ while cytokines such as IL-6,^54^ MCP1 and TNF-α have been found to be increased in COVID-19 virus positive patients.^73^ IL-6 has an atherogenic role and medium-to-long-term cardiovascular consequences may be caused by the increased IL-6 signalling.^33^ The inactivation of nitric oxide in vascular endothelium and the further production of arachidonic acid derivatives can further complicate a pre-existing artery disease.^77^ In addition, the suggestion of endothelial wall inflammation and endothelitis as shown in post-mortem studies,^85^ comes to support the possibility of endothelial dysfunction development.

The same principles exist for individuals who have undergone revascularization procedures before or at the era of COVID-19. Two articles have described the thrombotic complications of COVID-19 in patients who underwent either open or endovascular revascularization. Lacour et al reported thrombosis of a coronary artery stent which was implanted for acute myocardial infarction in a COVID-19 patient just 2 hours after angioplasty and they related this complication to the increased thrombotic burden the virus triggers.^92^ Giacomelli et al reported a bilateral acute limb ischaemia in a patient who had undergone open bifurcated aortic graft implantation for abdominal aortic aneurysm 6 years ago and was COVID-19 positive.^93^

Additionally, introduction of lockdown has led to the restriction of vascular services offered and the closure of outpatient activities worldwide, with a subsequent poorer surveillance of vascular patients, and thereby endangering limb integrity since timely diagnosis and management of critical limb ischaemia was not always feasible. Furthermore, patients themselves frightened by the contagious character of the virus, abstained from referring to vascular surgery services, with further deterioration of their vascular status. We expect with great interest the results of the COVER Study (COvid-19 Vascular sERvice Study).

Regarding chronic venous diseases and especially pre-existing DVT, we assume that an infection with COVID-19 can potentially deteriorate the clinical picture or create epithrombus or even new thrombosis in other sites, especially in individuals with underlying coagulation diseases. The thrombotic burden of the virus is increased and many of the thrombotic complications were experienced in individuals who already were under anti-coagulation treatment.^57,64^ Middeldorp et al reported that all the venous thromboembolism cases were diagnosed in patients receiving thrombosis prophylaxis.^57^ For patients implanted with venous stents we can deduce that they are at risk for in-stent thrombosis as well, taking into consideration the increased thrombotic burden in COVID-19. Although there are not available data yet, we can assume that chronic venous insufficiency can also be complicated with thrombosis in patients with COVID-19 viral infection.

The surveillance of patients with pre-existing thrombosis as well of those with chronic venous diseases with or without ulcers has also been restricted during the COVID-19 era in many centres. Therefore, we expect an increase of cases requiring treatment in the following months.

Currently there is an urgency to identify the optimal therapeutic agents that could be used in the management of COVID-19 cases. Some of them that have also been administered in the clinical setting can cause cardiovascular complications or alter the lipidemic profile of the patient.^33^ Guzik et al reported that after tocilizumab treatment serum levels of total cholesterol, high density (HDL) and low density lipoproteins (LDL) were increased, while cardiovascular markers such as HDL- serum amyloid A (SAA), phospholipase A2 and lipoprotein (α) were significantly reduced; lopinavir/ritonavir and interferon A2B can induce hypertriglyceridemia and hyperlipidemia.^33^ In addition, lopinavir/ritonavir result in decreased serum concentrations of active metabolites of clopidogrel and prasugrel and increased serum concentrations of ticagrelor.^82^ Therefore, the use of statins and antiplatelets should be balanced and personalized in the individuals who receive any of the aforementioned agents. Furthermore, chloroquine and hydroxychloroquine prolong QT interval and may therefore induce arrhythmias.^94^

However, the anti-inflammatory action of statins could have beneficial effect on the virus infection. Post hoc analysis of the JUPITER (Justification for the Use of Statins in Primary Prevention: An Intervention Trial Evaluating Rosuvastatin) trial observed a reduction in incident pneumonia with rosuvastatin.^95^ Parallel to this the effect of statin on atherosclerosis can potentially diminish or delay the long-term effects of COVID-19 virus on vascular pathology.^96^

COVID-19 has emerged only a few months ago and it is too premature to predict the long-term effects on the vascular system. Some assumptions can be performed based on the current literature data, the clinical implications and the prior experience with SARS-CoV virus, which shares similarity with SARS-CoV-2 virus.^97^ It was reported that patients who survived the infection suffered from long-term effects, as 68% of them developed abnormalities in lipid metabolism, and at 12 years follow-up cardiovascular abnormalities were present in 40% and altered glucose metabolism in 60%.^49^

## Conclusion

COVID-19 virus has dramatically changed the healthcare services worldwide, while its accurate impact cannot be still determined. It has affected particularly the elderly, while it has evolved through different clinical presentations. Cardiovascular disease is a risk factor for severe COVID-19 and poorer outcomes. However, there is no clear evidence for direct deterioration of existing peripheral artery disease. The disturbances in the coagulation mechanisms and the effects on vascular endothelium will provoke a surge of vascular complications in the coming months.

## Limitations

A sufficient number of the articles retrieved are still ahead of print, so there may be some information that could be re-evaluated or disproved and then published in the final form. Letters to the editor, interviews and conclusions from online presentations have not been included in this review.
